# Prevalence of chromosomal alterations in first-trimester spontaneous pregnancy loss

**DOI:** 10.1038/s41591-023-02645-5

**Published:** 2023-11-23

**Authors:** Rick Essers, Igor N. Lebedev, Ants Kurg, Elizaveta A. Fonova, Servi J. C. Stevens, Rebekka M. Koeck, Ulrike von Rango, Lloyd Brandts, Spyridon Panagiotis Deligiannis, Tatyana V. Nikitina, Elena A. Sazhenova, Ekaterina N. Tolmacheva, Anna A. Kashevarova, Dmitry A. Fedotov, Viktoria V. Demeneva, Daria I. Zhigalina, Gleb V. Drozdov, Salwan Al-Nasiry, Merryn V. E. Macville, Arthur van den Wijngaard, Jos Dreesen, Aimee Paulussen, Alexander Hoischen, Han G. Brunner, Andres Salumets, Masoud Zamani Esteki

**Affiliations:** 1 Department of Clinical Genetics, Maastricht University Medical Center (MUMC+), Maastricht, The Netherlands; 2 Department of Genetics and Cell Biology, GROW-Research Institute for Oncology and Reproduction, Faculty of Health, Medicine and Life Sciences (FHML), Maastricht University, Maastricht, The Netherlands; 3 Research Institute of Medical Genetics, Tomsk National Research Medical Center of the Russian Academy of Sciences, Tomsk, Russia; 4 Department of Biotechnology, Institute of Molecular and Cell Biology, University of Tartu, Tartu, Estonia; 5 Department of Anatomy & Embryology, Faculty of Health, Medicine and Life Sciences (FHML), Maastricht University, Maastricht, The Netherlands; 6 Department of Clinical Epidemiology and Medical Technology Assessment (KEMTA), Maastricht University Medical Center (MUMC+), Maastricht, The Netherlands; 7 Department of Obstetrics and Gynaecology, Institute of Clinical Medicine, University of Tartu, Tartu, Estonia; 8 Department of Obstetrics and Gynecology, University of Helsinki, Helsinki, Finland; 9 Department of Obstetrics and Gynaecology, Maastricht University Medical Center (MUMC+), Maastricht, The Netherlands; 10 Department of Human Genetics, Radboud University Medical Center, Nijmegen, The Netherlands; 11 Department of Internal Medicine, Center for Infectious Disease (RCI), Radboud University Medical Center, Nijmegen, The Netherlands; 12 Radboud Institute for Molecular Life Sciences, Radboud University Medical Center, Nijmegen, The Netherlands; 13 Radboud Expertise Center for Immunodeficiency and Autoinflammation and Radboud Center for Infectious Disease (RCI), Radboud University Medical Center, Nijmegen, The Netherlands; 14 Competence Center on Health Technologies, Tartu, Estonia; 15 Division of Obstetrics and Gynecology, Department of Clinical Science, Intervention & Technology (CLINTEC), Karolinska Institutet and Karolinska University Hospital, Stockholm, Sweden

**Keywords:** Mosaicism, Genetics research

## Abstract

Pregnancy loss is often caused by chromosomal abnormalities of the conceptus. The prevalence of these abnormalities and the allocation of (ab)normal cells in embryonic and placental lineages during intrauterine development remain elusive. In this study, we analyzed 1,745 spontaneous pregnancy losses and found that roughly half (50.4%) of the products of conception (POCs) were karyotypically abnormal, with maternal and paternal age independently contributing to the increased genomic aberration rate. We applied genome haplarithmisis to a subset of 94 pregnancy losses with normal parental and POC karyotypes. Genotyping of parental DNA as well as POC extra-embryonic mesoderm and chorionic villi DNA, representing embryonic and trophoblastic tissues, enabled characterization of the genomic landscape of both lineages. Of these pregnancy losses, 35.1% had chromosomal aberrations not previously detected by karyotyping, increasing the rate of aberrations of pregnancy losses to 67.8% by extrapolation. In contrast to viable pregnancies where mosaic chromosomal abnormalities are often restricted to chorionic villi, such as confined placental mosaicism, we found a higher degree of mosaic chromosomal imbalances in extra-embryonic mesoderm rather than chorionic villi. Our results stress the importance of scrutinizing the full allelic architecture of genomic abnormalities in pregnancy loss to improve clinical management and basic research of this devastating condition.

## Abstract (web summary)

Genomic analysis of products of conception collected after spontaneous pregnancy loss in the first trimester reveals previously undetected chromosomal aberrations and a higher degree of mosaic chromosomal imbalances.

## Main

Worldwide, 23 million pregnancy losses occur every year, with a high prevalence of 10–15% of all clinically recognized pregnancies^1^. Pregnancy loss primarily occurs before weeks 8–9 of gestation^1^, and there is considerable additional loss in earlier stages of pregnancy that may go unnoticed. Overall, 10.8% of women experience at least one pregnancy loss, and 1.9% and 0.7% have two or three pregnancy losses, respectively^1^. Identifying the cause of pregnancy loss can provide important prognostic, diagnostic and management recommendations to support future viable pregnancies^2^. Chromosomal abnormalities, in particular aneuploidy, defined as an incorrect number of chromosomes, in the conceptus are the leading causes of pregnancy losses. It has been established that aneuploidies commonly occur during oogenesis^3–5^ and in early embryogenesis^6–8^. The incidence of chromosomal aneuploidies increases with maternal age, which contributes to age-related infertility^8^. This is due to the low fidelity of chromosome segregation in meiosis during oogenesis^3,8^ and DNA replication stress^9^ during mitotic cleavage divisions in pre-implantation embryogenesis^6,7^. Previously, we and others demonstrated that, although chromosome instability (CIN) is common in early embryogenesis^6,7^, it is not present at birth^10^. This observation indicates that only embryos with sufficient genome integrity can survive to term and that both meiotic aneuploidies in oocytes and post-zygotic chromosome abnormalities in early embryogenesis may lead to implantation failure and pregnancy losses^1^.

CIN leads to mosaic embryos that contain both chromosomally normal and abnormal cells. It has been shown that aneuploid cells in mosaic embryos can be progressively depleted during pre-implantation development^11^. Self-correction of human embryos may operate via cellular fragmentation and blastomere exclusion of abnormal cells^12^ or by rescue mechanisms, such as trisomy or monosomy rescue^13^. Spatiotemporal allocation of abnormal cells or aneuploidy rescue mechanisms can lead to confined placental mosaicism, which is the main biological cause for discordant abnormal non-invasive prenatal testing (NIPT) results, with normal fetus confirmed after invasive fetal testing^14^. For instance, we previously showed that more than 70% of large (>100 kilobase (kb)) de novo copy number variations (CNVs) are present only in the placental lineage^10^.

The genomic landscape of second-trimester and third-trimester pregnancy losses and elective terminations of fetuses with abnormal in utero phenotypes has been characterized^15^. However, little is known about the genomic landscape of first-trimester spontaneous pregnancy losses. This knowledge is essential to understanding in utero mechanisms of CIN separately for fetal and placental lineages and to developing strategies for the early detection of high-risk pregnancies leading to pregnancy loss. In this study, we profiled the chromosomal landscape of the chorionic villi and the extra-embryonic mesoderm of first-trimester (~7 weeks of gestational age) spontaneous pregnancy losses, which are derived from the embryonic trophectoderm and the inner cell mass, respectively.

## Results

### Cohort characteristics and conventional cytogenetic tests

**Fig. 1:**
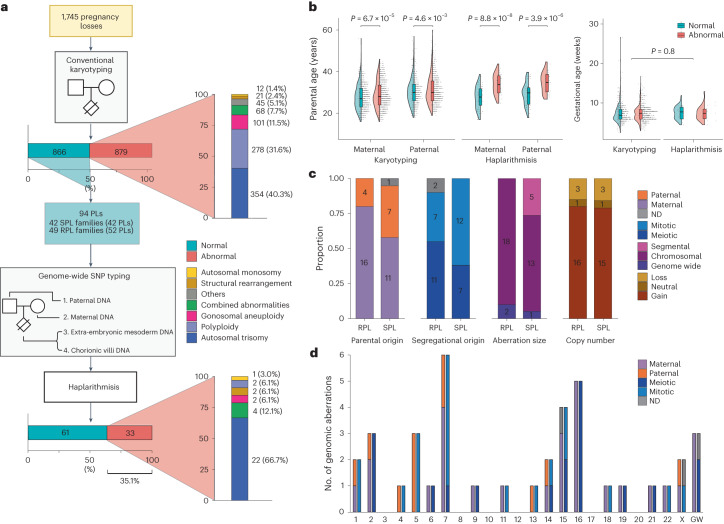
Genome haplarithmisis reveals previously undetected chromosomal aberrations. **a**, Study design and distribution of aberrations. **b**, Maternal, paternal and gestational age in conventional karyotyping of POC samples with normal (*n* = 866) and abnormal (*n* = 879) karyotypes and in genome haplarithmisis POC samples with normal (*n* = 61) and abnormal (*n* = 33) genomes (two-sided Welch’s *t*-test). The box plot represents the 25th percentile, median and 75th percentile, respectively, and the whiskers extend to the farthest data point that is no more than 1.5 times the interquartile range (IQR) from the upper or lower quartile. **c**, Parental and segregational origin of aberrations, aberration size (segmental, chromosomal and genome wide) and copy number (gain, loss and neutral) of unique aberrations per POC sample for the RPL cohort (*n* = 20) and the SPL cohort (*n* = 19). **d**, Parental and segregational origin of genomic aberration per chromosome, including each unique aberration per POC sample. PL, pregnancy loss. ND, not determined; POC, product of conception; SPL, sporadic pregnancy loss; RPL, recurrent pregnancy loss. Source data

After conventional karyotyping of the products of conception (POCs) that were collected from 1,745 women over a course of 35 years (1987–2021), 866 (49.6%) and 879 (50.4%) pregnancy losses were classified as karyotypically normal and abnormal, respectively (Fig. 1a and Table 1). Of the 1,745 cases, 1,597 (91.5%) samples were karyotyped using conventional GTG banding after long-term extra-embryonic fibroblast culture, and 29 (1.7%) samples were karyotyped by direct preparations of the chorionic villi. If GTG banding was not possible, other traditional methods, including conventional comparative genomic hybridization (CGH) (3.4%, 59 samples) and interphase fluorescence in situ hybridization (FISH) with centromere enumeration probes (3.4%, 60 samples), were performed.

**Table 1:** Clinical diagnosis of early pregnancy loss

Diagnosis	Conventional karyotyping		Genome haplarithmisis	
	*n*	%	*n*	%
Missed abortions	1,156	66.2	74	78.7
Anembryonic pregnancies	261	15.0	16	17.0
Spontaneous abortions	137	7.9	1	1.1
Fetus malformations	27	1.5	0	0
Hydatidiform mole	4	0.2	0	0
Inconclusive pregnancy loss etiology	160	9.2	3	3.2
Total POCs	1,745	100	94	100

In line with previous studies^1,16,17^, abnormal fetal karyotypes were associated with higher parental age (maternal age: 29.0 ± 6.4 s.d. and 27.8 ± 5.9 s.d., respectively, *P* = 6.7 × 10^−5^; paternal age: 31.3 ± 6.8 s.d. and 30.3 ± 6.3 s.d., respectively, *P* = 4.6 × 10^−3^; two-sided Welch’s *t*-test) (Fig. 1b). Logistic regression was performed to further investigate whether maternal and paternal age were independently associated with abnormal POC karyotypes. Implementing both parental ages in the same regression model dissolved the statistically significant association for both factors, indicating that maternal and paternal age separately explain the same variance in the data and show high collinearity (Supplementary Table 1).

### Genomic alterations in karyotypically normal POCs

**Fig. 2:**
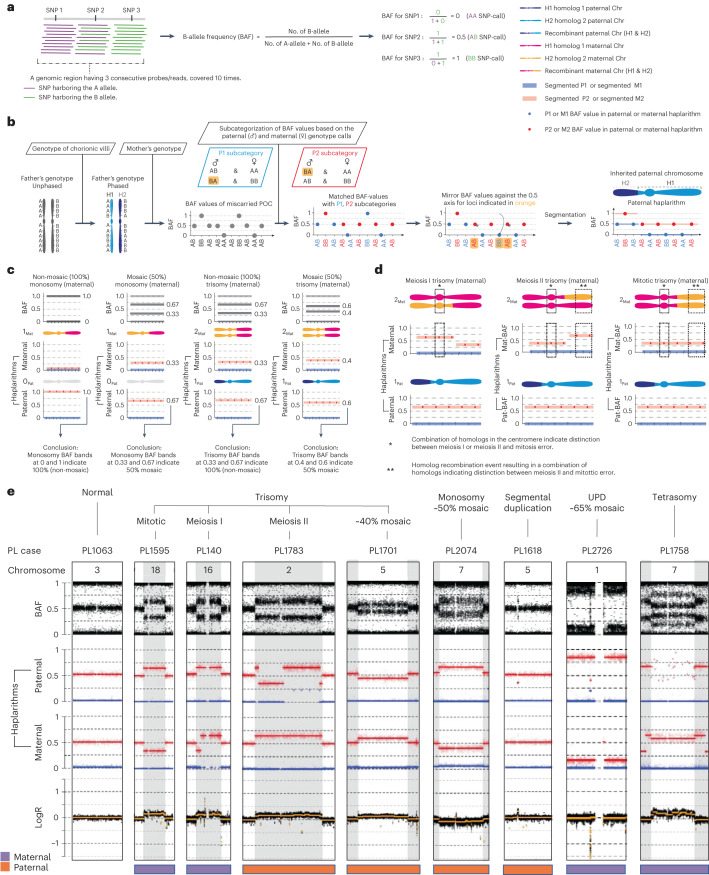
Schematic representation of genome haplarithmisis and detection of various abnormalities. **a**, A genomic region harboring three consecutive SNPs, each with weighted signal intensity of 10, as well as equation for BAF computation for those three SNPs. **b**, Schematic representation of the standard genome haplarithmisis workflow as demonstrated in Zamani Esteki et al.^7^. Detection of different levels of mosaicism in trio-based haplarithmisis (**c**) and parental and segregational origin of genomic anomalies in trio-based haplarithmisis (**d**) (Methods). **e**, Haplarithms of several pregnancy losses with different types of abnormalities, parental and segregational origins and mosaicism degrees; only a single chromosome of interest is displayed per POC sample. PL1063 has a normal diploid Chr 3; PL1595 has a non-mosaic trisomy 18 of maternal and mitotic error origin; PL140 has a non-mosaic trisomy 16 of maternal and meiosis I error origin; PL1783 has a non-mosaic trisomy 2 of paternal and meiosis II error origin; PL1701 has an approximately 40% mosaic trisomy 5 of paternal and mitotic error origin; PL2074 has an approximately 50% mosaic monosomy 7 of paternal (maternal chromosome is left) and mitotic error origin; PL1618 has a (subchromosomal) approximately 2.7-Mb duplication in Chr 5 near the centromere (q11.1–q11.2) of paternal and mitotic error origin; PL2726 has an approximately 65% mosaic UPD 1 of maternal and mitotic origin; and PL1758 has a tetrasomy 7 of maternal error origin (Extended Data Fig. 7). PL, pregnancy loss.

**Extended Data Fig. 1:**
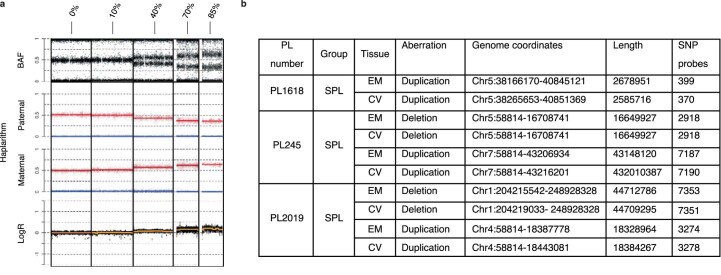
Mosaicism of > 10% and CNVs > 100 kb are detected by genome haplarithmisis. **a**, Different mosaicism degrees for chromosomal trisomy of paternal origin of several PLs. **b**, Detected CNVs for 3 PLs including genome coordinates, length, and number of SNP probes.

We analyzed 94 karyotypically normal pregnancy losses with good-quality DNA samples (Methods) from 91 families with similar gestational ages as the entire cohort (7.5 ± 2.2 s.d. and 7.5 ± 1.7 s.d. gestational weeks, respectively, *P* = 0.8, two-sided Welch’s *t*-test) (Fig. 1b and Methods). These samples were selected based on (1) their classification as ‘normal’ by conventional karyotyping; (2) the availability of POC extra-embryonic mesoderm and chorionic villi tissues and parental DNA; and (3) parents who were were karyotypically normal, without genetic predisposition for pregnancy loss. To detect (mosaic) de novo genomic aberrations in POCs undetected by conventional karyotyping, we performed genome-wide single-nucleotide polymorphism (SNP) genotyping of parental as well as extra-embryonic mesoderm and chorionic villi DNA from POCs, followed by genome haplarithmisis^7,10^. Haplarithmisis is a conceptual method that transforms genotyping data to haplotypes and copy number states, called parental haplarithms. When a copy number change affects a combination of different homologous chromosomes of a parent, this represents meiotic error. If the centromere is from different homologous chromosomes, this represents meiotic I error. If the centromere is not involved, but a part of the chromosome derives from different homologous chromosomes, this specifies meiotic II error^18^. In addition, distortion of B-allele frequency (BAF) values from expected 1_maternal_:1_paternal_ allelic ratio indicates the degree (%) of abnormal cells—that is, mosaicism^10,19^. Here, we applied haplarithmisis on bulk DNA samples (that is, derived from many cells) and made use of chorionic villi as a seed to phase the parental genomes, allowing the reconstruction of trio-based parental haplarithms (Fig. 2a and Methods). This allowed us to determine the prevalence and nature of different chromosomal abnormalities, including their parental and mechanistic origins (Figs. 1c,d and 2a,b) and their levels of mosaicism (Fig. 2a,b and Extended Data Fig. 1). The data showed that, of 94 POCs (188 paired chorionic villi and extra-embryonic mesoderm DNA samples; 89 families with a single pregnancy loss, one family with two pregnancy losses and one family with three pregnancy losses), 65 DNA samples (34.6%; 33 chorionic villi and 32 extra-embryonic mesoderm DNA samples) had a genomic aberration (Source Data). Thus, out of 94 karyotypically normal POC samples, as determined by conventional analysis, 33 POC samples (35.1%) had one or more genomic imbalances that were detected by genome haplarithmisis (Fig. 1a and Supplementary Table 2). If we consider these haplarithmisis-determined abnormal samples (35.1%) as well as the 50.4% abnormality rate reported through conventional karyotyping (*n* = 879/1,745), the rate of genomic aberrations in POC samples reaches 67.8% by extrapolation (Methods). This is higher than what has been quoted previously by other studies that used karyotyping^13,20^ or clinical-grade chromosomal microarrays^21–29^ (Supplementary Table 3).

### Profiling the genomic landscape of sporadic and recurrent pregnancy losses

**Extended Data Fig. 2:**
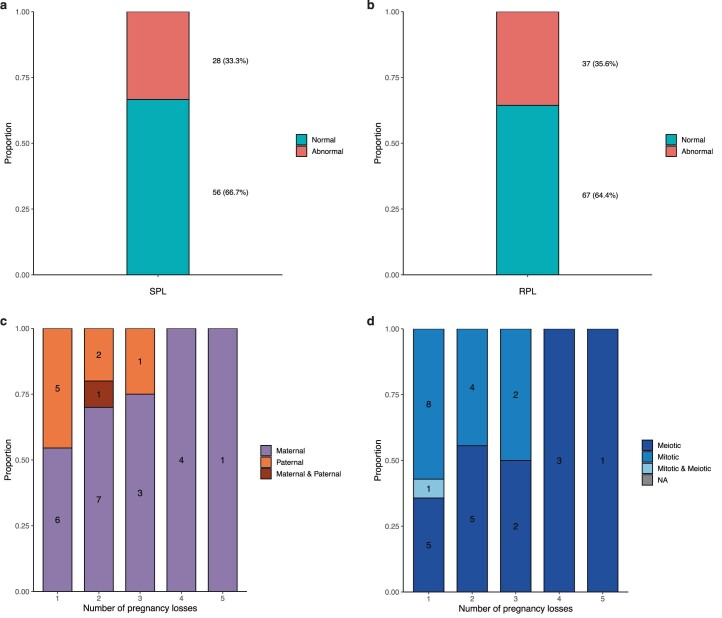
Abnormality rate per tissue between SPL and RPL with parental and segregational origin. **a**, Abnormality rate of SPL DNA samples (n = 84). **b**, Abnormality rate of RPL DNA samples (n = 104). **c**, Parental origin and number of PLs per family (n = 30). **d**, Segregational origin and number of PLs per family (n = 32).

To further characterize the genetic effect of the detected aberrations, we divided the 94 pregnancy losses into sporadic pregnancy loss (SPL), defined as one pregnancy loss (42 families and pregnancy losses, 84 DNA samples), and recurrent pregnancy loss (RPL), defined as two or more consecutive pregnancy losses (49 families, 52 pregnancy losses, 104 DNA samples) (Fig. 1a). The SPL and RPL cohorts had 28 (33.3%, 19 unique copy number aberrations per POC) and 37 (35.6%, 20 unique copy number aberrations per POC) POC samples with genomic aberrations, respectively (Extended Data Fig. 2). The RPL and SPL cohorts did not show a significant difference in either segregational and parental origins of aberrations or in the total copy number or copy-neutral events (Fig. 1c). However, the SPL cohort contained more segmental aberrations, whereas the RPL cohort contained more numerical chromosomal aberrations (Fig. 1c; *P* = 3.6 × 10^−2^, Fisher’s exact test).

Aberrations on Chr 7 and Chr 16 were most common in first-trimester pregnancy loss (Fig. 1d), as observed previously^1,30^. Aberrations on Chr 16 (*n* = 5) were all of maternal and meiotic in origin, and aberrations on Chr 7 were maternal and mitotic (*n* = 4), paternal and mitotic (*n* = 1) and paternal and meiotic (*n* = 1) in origin (Fig. 1d). Trisomy 16 impairs embryonic growth due to placental hyperplasia, potentially leading to first-trimester pregnancy loss^30^. Trisomy 16 is less prevalent in NIPT samples at 11–12 weeks of pregnancy^31,32^ as compared to pre-implantation embryos after pre-implantation genetic testing (PGT)^33,34^, indicating that pregnancies with trisomy 16 may have reduced capacity to reach to later gestational ages.

### Determining the level of mosaicism in chorionic villi and extra-embryonic mesoderm tissues

**Fig. 3:**
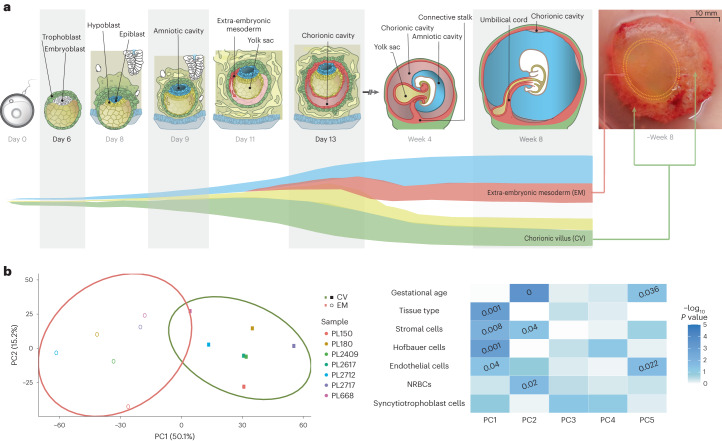
Extra-embryonic mesoderm and chorionic villi lineages and their cellular composition. **a**, Schematic representation of early embryonic development. Extra-embryonic mesoderm (EM) develops from the embryoblast lineage (hypoblast and epiblast), whereas chorionic villi (CV) develop from the trophoblast lineage. EM and CV samples from pregnancy losses were collected at week 7.6 ± 1.7 s.d. **b**, PCA of all CpG sites (*n* = 685,221) passing quality control criteria (Methods) in data from high-DNA-quality EM (*n* = 6) and CV (*n* = 7) samples. The ellipses represent the 90% confidence interval, and the percentage of variance explained by each principal component (PC) is shown in brackets. Heat map showing associations between the first five PCs and biological aspects of the samples, including their predicted cell compositions (Extended Data Fig. 6). The color gradient shows the −log_10_ of the *P* values, and *P* values less than 0.05 are indicated. The significance of the correlation between the PCs and the continuous, numerical sample attributes was tested using a permutation test with 10,000 permutations. The association between the PCs and the binary variable tissue type was assessed using a two-sided Wilcoxon signed-rank test.

**Extended Data Fig. 3:**
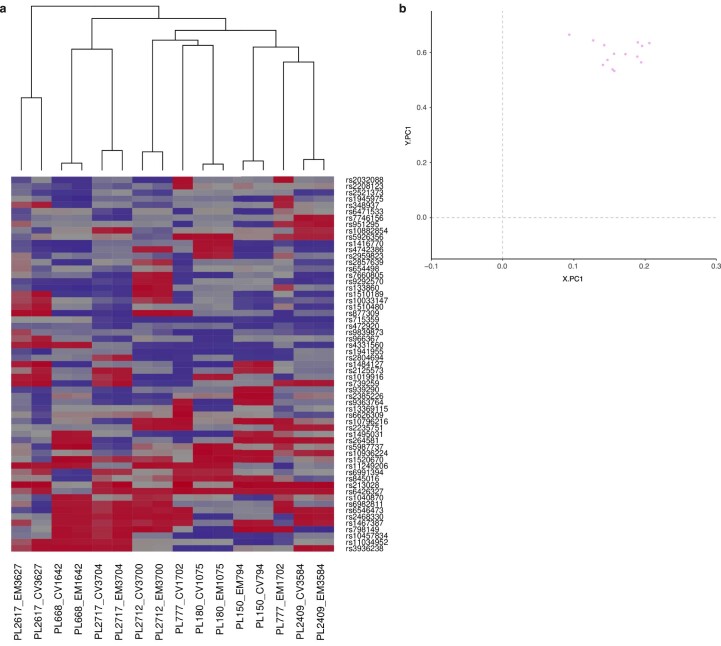
Heatmap and PCA of EM and CV tissue samples. **a**, Heatmap of EPIC array SNP probes (n = 59) of all paired EM and CV tissue samples (n = 13). Hierarchical clustering of the samples was constructed using Euclidian distance and complete linkage and is shown as a dendrogram. **b**, Scatter plot showing the projection of all EM and CV tissue samples (n = 13) into the principal component space generated using reference data for sex prediction (not shown). All chosen samples were female according to the haplotyping analysis and the methylation sex prediction. Grey dashed lines separate the quadrants where male (XY) samples are expected to map to the lower left quadrant and female (XX) samples to the upper right quadrant.

**Extended Data Fig. 4:**
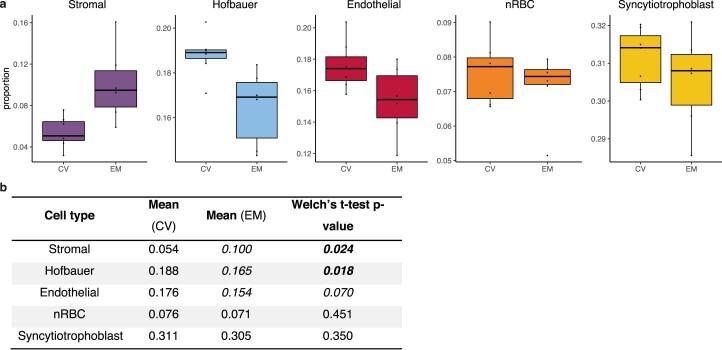
Predicted cell composition of EM and CV samples based on methylation data. **a**, Boxplots showing the predicted cellular composition of the high DNA quality EM (n = 6) and CV (n = 7) samples for each cell type: stromal cells, Hofbauer cells, Endothelial cells, nucleated red blood cells (nRBCs) and syncytiotrophoblast cells. The horizontal lines of the of the boxplot represent the 25^th^ percentile, median and 75^th^ percentile respectively while the whiskers extend to the farthest data point that is no more than 1.5 times the interquartile range (IQR) from the upper or lower quartile. The dots represent individual samples. **b**, Table with mean values of each cell type, P values were calculated with two-sided Welch’s T-test.

Comparing the haplarithm profiles of extra-embryonic mesoderm and chorionic villi allowed us to determine not only if these tissues carry different large CNVs (>100 kb) but also whether the level of mosaicism is different. The analysis of extra-embryonic mesoderm and chorionic villi DNA samples can be used to probe the spatiotemporal allocation of abnormal cells in early embryogenesis and to deduce whether this allocation affects the fate of early prenatal development and the risk of pregnancy loss. To check for accurate dissection of extra-embryonic mesoderm and chorionic villi tissues, we performed methylome-wide analysis of 13 samples that generated good-quality data (Extended Data Fig. 3 and Methods). Principal component analysis (PCA) of all methylation sites showed clear separation of chorionic villi and extra-embryonic mesoderm tissues (Fig. 3b). Subsequent cell composition deconvolution suggested that both chorionic villi and extra-embryonic mesoderm samples were of mixed, but different, cellular origin, with a significantly higher proportion of Hofbauer cells as well as a significantly lower proportion of stromal cells in chorionic villi samples compared to the extra-embryonic mesoderm samples (Extended Data Fig. 4).

**Fig. 4:**
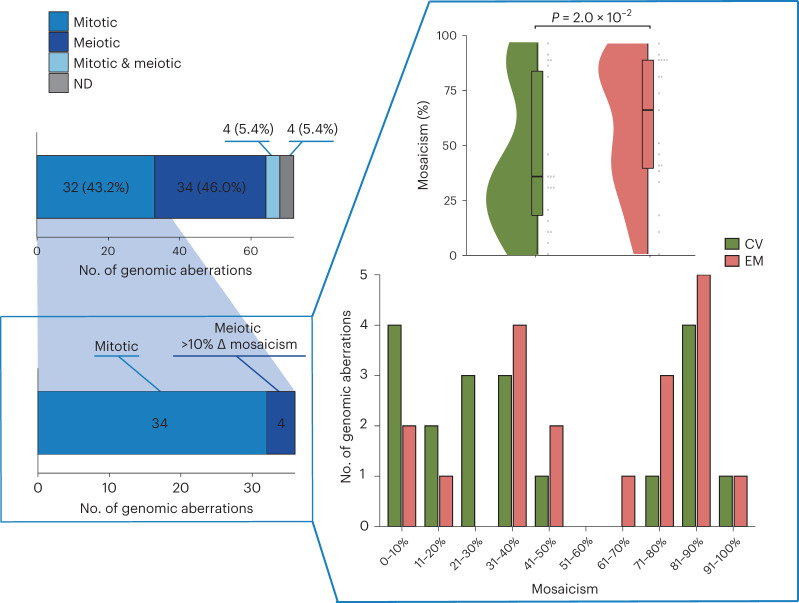
Segregational and lineage origins of pregnancy losses. Segregational origin of each individual aberration in both extra-embryonic mesoderm (EM) and chorionic villi (CV) tissue per POC detected by genome haplarithmisis, whereas samples with aberrations of mitotic origin (*n* = 34: 17 EM and 17 CV) or meiotic origin with greater than 10% mosaicism difference between EM and CV (*n* = 4: two EM and two CV) were selected for EM/CV plots and mosaicism statistics. The mosaicism degree in EM samples (*n* = 19) was significantly higher than that in CV samples (*n* = 19) (two-sided Wilcoxon signed-rank test). The box plot represents the 25th percentile, median and 75th percentile, respectively, whereas the whiskers extend to the farthest data point that is no more than 1.5 times the interquartile range (IQR) from the upper or lower quartile. ND, not determined.

**Fig. 5:**
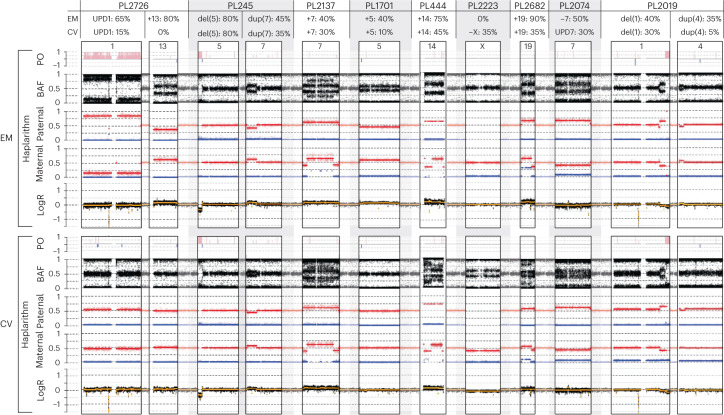
Degree of mosaicism in extra-embryonic mesoderm as compared to chorionic villi. Haplarithms of nine pregnancy loss samples with differences between extra-embryonic mesoderm (EM) and chorionic villi (CV) of greater than 10% mosaicism. PL2726 has a (EM ~65%, CV ~15%) mosaic UPD 1 of maternal and mitotic origin and an approximately 80% mosaic EM-only trisomy 13 of paternal and mitotic error origin. PL245 has a (EM ~80%, CV ~80%) mosaic (subchromosomal) approximately 16.7-Mb deletion in Chr 5 (p15.1–p15.33) of paternal and mitotic error origin and a (EM ~45%, CV ~35%) mosaic approximately 43.2-Mb duplication in Chr 7 (p14.1–p22.3) of paternal and mitotic error origin. PL2137 has a (EM ~40%, CV ~30%) mosaic trisomy 7 of maternal and mitotic error origin. PL1701 has a (EM ~40%, CV ~10%) mosaic trisomy 5 of paternal and mitotic origin. PL444 has a (EM ~75%, CV ~45%) mosaic trisomy 14 of maternal and meiotic error origin. PL2223 has an approximately 35% mosaic CV-only monosomy X of paternal error origin. PL2682 has a (EM ~90%, CV ~35%) mosaic trisomy 19 of maternal meiotic error origin. PL2074 has an approximately 50% mosaic EM-only monosomy 7 of maternal and mitotic error origin and an approximately 30% mosaic CV-only UPD 7 of maternal and mitotic error origin. PL2019 has a (EM ~40%, CV ~30%) mosaic approximately 44.7-Mb deletion in Chr 1 (q32.1–q44) of paternal and mitotic error origin and a (EM ~35%, CV ~5%) mosaic approximately 18.3-Mb duplication in Chr 4 (p15.32–p16.3) of paternal and mitotic error origin. del, deletion; dup, duplication; Mb, megabase; +, trisomy; −, monosomy; PO, parent-of-origin.

**Extended Data Fig. 5:**
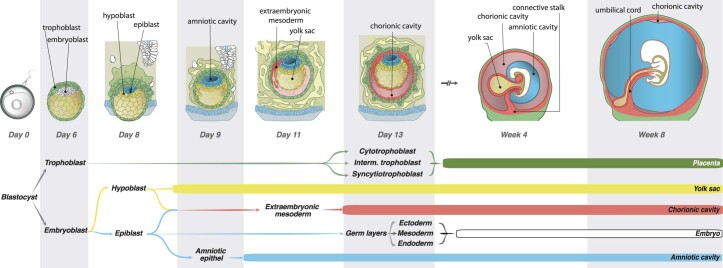
Detailed schematic representation of early embryonic development. Trophoblast and embryoblast lineages develop at day 6, hypoblast and epiblast develop from the embryoblast at day 8, hypoblast develops into the yolk sac, while epiblast develops into the different embryonic germ layers and the embryo and into the amnionic cavity. Extraembryonic mesoderm develops around day 11 from the hypo- and epiblast. The trophoblast cells become the placenta including cytotrophoblast, intermediate trophoblast, and syncytiotrophoblast cells, chorionic villi develop around day 13 from the trophoblast lineage (see also Fig. 2a).

Although there is no doubt that the chorionic villi are derived from the trophectoderm^35,36^, there is some uncertainty surrounding the developmental origin of extra-embryonic mesoderm^35,36^. A recent study proposed that the extra-embryonic mesoderm develops from the hypoblast-derived primary yolk sac, supplemented by epiblast-derived mesoderm from the gastrulating embryo^35^ (Fig. 3a and Extended Data Fig. 5). Hypoblasts and epiblasts are both derived from the inner cell mass. Of the 33 genetically aberrant POC samples, a selection consisting of all tissues with a mitotic aberration or meiotic aberration with a greater than 10% difference in the proportion of abnormal cells comparing extra-embryonic mesoderm and chorionic villi showed that the extra-embryonic mesoderm biopsies had a higher level of mosaicism relative to chorionic villi (58.2% ± 30.1 s.d. and 43.4% ± 32.9 s.d., respectively, *P* = 2.0 × 10^−2^, Wilcoxon signed-rank test; Fig. 4). Of the nine POC samples (27.3%) with a greater than 10% difference in level of mosaicism, eight samples (six aberrations of mitotic origin and two of meiotic origin) had aberrations in autosomes and one sample (aberration of mitotic origin) in Chr X (Fig. 5). In all samples with autosomal aberrations, the level of mosaicism was higher in extra-embryonic mesoderm than in chorionic villi (Figs. 4 and 5). This contrasts with viable pregnancies where mosaic abnormalities are often restricted to the chorionic villi^35,36^. For two cases, copy number aberrations (trisomy 13 of PL2726 and monosomy 7 of PL2074; Fig. 5 and Extended Data Fig. 5) were detected only in extra-embryonic mesoderm, with post-zygotic mitotic origin. These support the theoretical model for tissue-specific aneuploid cell line compartmentalization in early pregnancy loss^13^.

## Discussion

Even though CIN is common in early embryogenesis and leads to mosaic embryos carrying both chromosomally normal and abnormal cells^6,7^, the abnormal cells are not predominantly present at birth^10^. However, the effect of post-zygotic CIN on first-trimester prenatal development is not clear. In this study, we carried out parallel analysis of extra-embryonic mesoderm and chorionic villi samples from the same POCs, increasing the diagnostic yield of detecting genomic aberrations. Strikingly, mosaicism tended to be higher in extra-embryonic mesoderm relative to chorionic villi, which again suggests persistent involvement of abnormal fetal cells in pregnancy loss. Over 90% of pregnancy losses occur during the first trimester^37,38^. Chromosomal abnormalities in the fetus are recognized as a primary cause of pregnancy loss. Previously, eight large studies (>1,000 POC sample size, in total 42,500 POCs) showed a combined yield of approximately 53.7% fetal chromosomal abnormalities^21–28^ (Supplementary Table 3), which is in line with the 53.1% reported rate in a recent meta-analysis^29^. The study that applied high-density SNP arrays reported a higher yield of 67%, which is similar to what we found here, in the present study, after genome haplarithmisis^26^. Thus, haplarithmisis gives a superior yield over karyotyping or standard microarray approach. In addition, six previous studies analyzed POCs that were classified as normal with conventional karyotyping^39–44^ (Supplementary Table 4). The average frequency of additionally detected abnormalities found in these studies was 19.4%, which is nearly two-fold lower than the 35.1% identified in the present study. This difference can be explained by two major factors. First, the previous studies relied on single POC tissue analysis, primarily chorionic villi or placenta. Second, the conventional cytogenetic methods, including microarrays, are unable to distinguish the meiotic and mitotic origins of genomic aberrations and detect low-level mosaicism. Here we demonstrate that these shortcomings can be tackled using genome haplarithmisis.

**Extended Data Fig. 6:**
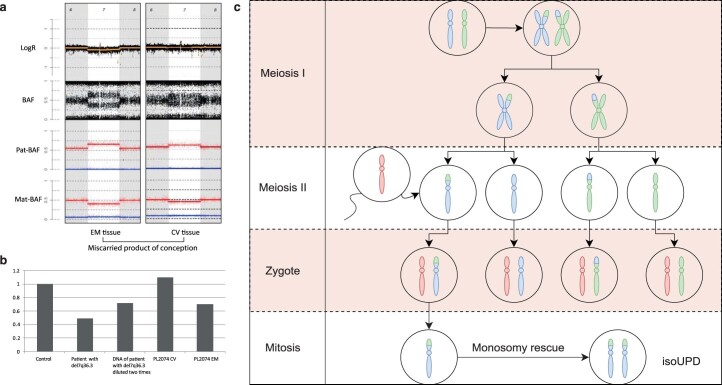
Validation of chromosome 7 copy number in PL2074. **a**, Haplarithm of EM and CV tissue of PL2074, EM shows a 50% mosaic monosomy 7 while CV shows a ~ 30% UPD (copy-neutral) with contamination of maternal tissue. **b**, RT-qPCR results with a diploid control, patient with a 7q36.3 deletion and two times diluted, PL2074 CV (UPD, copy neutral), and PL2074 ( ~ 50% mosaic monosomy 7) with contamination of maternal tissue. **c**, Schematic representation of proposed monosmy-rescue mechanism leading to uniparental disomy (UPD).

**Extended Data Fig. 7:**
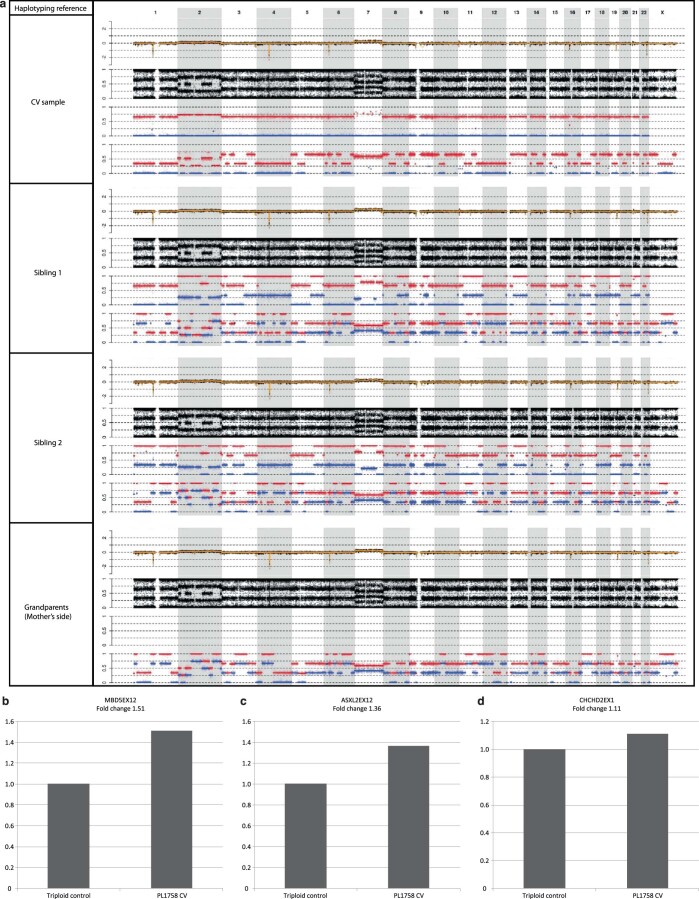
Validation of segregational origin of aberrations in PL1758 CV DNA sample. **a**, Haplarithms of fetal EM of PL1758, produced with different references for phasing, namely CV of the same fetus, 2 different siblings, and maternal grandparents. PL1758 shows complex abnormalities with genome-wide triploidy of maternal error origin, and mosaic tetrasomy of Chr 2 and Chr 7, segregational origin appear to be mitotic (Chrs 1, 4, 12), or meiotic II (Chrs 3, 5, 6, 8, 9, 10, 11, 13, 14, 15, 16, 17, 18, 19, 20, 21, 22). Haplarithmisis can accurately determine segregational origin with CV as reference compared to siblings or grandparents as a reference. Validation of PL1758 by RT-qPCR using DNA from spontaneous PL with 69,XXX karyotype and primers for **b**, exon 12 of the *MBD5* gene (2q23.1), **c**, exon 12 of the *ASXL2* gene (2p23.3), and **d**, exon 1 of the *CHCHD2* gene (7p11.2) were used to confirm tetrasomy for Chr 2 and Chr 7 in DNA from CV of PL1758.

The conventional karyotyping of POCs being applied in routine care is limited by its low resolution, contamination from maternal cells, high culture failure rates and overgrowth of (maternal) normal cells compared to abnormal cells, leading to low diagnostic yield^45^. The quality also varies between samples and laboratories and is reliant on the expertise of technicians and cytogeneticists. Previously, we and others showed that low proliferative activity of extra-embryonic cells in vitro is a major limitation of conventional karyotyping of spontaneous pregnancy losses. Specifically, conventional cytogenetic analysis of miscarriages strongly depends on tissue culturing and is associated with a substantial culture failure rate, which varies from 5% to 42% in different laboratories^46–55^. This suggests that the use of sophisticated genome analysis methods that use DNA samples and do not require cell culturing in pregnancy loss samples carry clear advantages. Genome haplarithmisis can detect low-grade mosaicism (>10%) from uncultured samples (Fig. 2a, Extended Data Fig. 1 and Methods) with higher resolution and allows for the detection of smaller subchromosomal CNVs (>100 kb) (Fig. 2e and Extended Data Fig. 1b)^10^. Additionally, it can detect the parental and segregational origins of aberrations and maternal cell contamination^7^ (Fig. 2d and Extended Data Figs. 6 and 7). These features are well beyond the sensitivity of conventional methods, such as karyotyping and standard chromosomal microarray-based or sequencing-based copy number analyses that are being performed in routine care. There is an emerging need for prospective clinical studies comparing genome haplarithmisis with conventional methods to evaluate its clinical implementation and cost-effectiveness for management of pregnancy loss. The guidelines of the European Society of Human Reproduction and Embryology (ESHRE) in RPL restated the limitation of conventional karyotyping and underscored the importance of future studies that explore the role of next-generation sequencing techniques^56^. A strategy using genetic analysis of miscarriage tissue could help patients deal with the psychological impact of pregnancy loss and would limit the need for further expensive and elaborate maternal investigations for other causes of RPL^57^.

Profiling the genomic landscape of pregnancy losses by carefully dissecting both the extra-embryonic mesoderm and chorionic villi tissues of the same POC and applying haplarithmisis allowed us to detect and classify 35.1% of karyotypically normal POCs as abnormal. Moreover, POCs with different levels of mosaicism in extra-embryonic mesoderm and chorionic villi were found, with the prevalence of aberrations being higher in extra-embryonic mesoderm compared to chorionic villi. This finding raises intriguing hypotheses about the origins of mosaic mutations in pregnancy loss. Although extra-embryonic mesoderm develops before gastrulation in primates, it develops during gastrulation in rodents. Therefore, extra-embryonic mesoderm cells are most likely derived from transient primary yolk sac, which is of embryoblast, and not trophoblast, origin^35^. It was previously suggested that self-correction mechanisms for aneuploidy are active in the inner cell mass during early embryogenesis and that there is a selective bottleneck in early embryogenesis when aneuploid cells are depleted^11,12^. Our data are compatible with a selective scenario in which chromosomal aberrations first emerge in the inner cell mass of the blastocyst and persist at least in extra-embryonic mesoderm, conferring a strong detrimental effect on the pregnancy outcome, which may result in pregnancy loss.

We found that SPL and RPL cohorts have similar prevalence of genomic aberrations. However, these two groups had different types of genomic aberrations, such that the SPL cohort had significantly more segmental aberrations, whereas the RPL cohort carried more aneuploidies (Fig. 1c). Although our cohort is underpowered to draw firm conclusions, our POC analysis points to possible differences in the genetic etiology of pregnancy losses, which merits further study. We emphasize the importance of reaching an international consensus on the clinical definition of RPL, which is the topic of an ongoing debate and differs across guidelines and countries^1,56^. ESHRE’s guidelines from 2022 define RPL as the loss of two or more pregnancies before 24 weeks of gestation^56^, which was used in the present study.

Our data inform discussions about the clinical importance of scrutinizing the full allelic architecture of genomic abnormalities and their segregational origin (meiotic versus mitotic origin) in human embryos and pregnancies with relevance for the safety of transferring in vitro fertilized (IVF) embryos with mosaic imbalances^58^ as well as interpretation of NIPT results with mosaic aberration indications. In nationwide NIPT studies^31,32^, the rate of confirmed confined fetal mosaicism is very low compared to confined placental mosaicism, indicating that abnormal cells in the fetus are less tolerated relative to the placenta. For instance, 94% of rare autosomal trisomies in NIPT were found to be most likely due to confined placental mosaicism^31^. The impact of chromosomal mosaicism is less clear due to current limitations for its detection. According to some rare cytogenetic studies on spontaneous pregnancy losses and confined placental chromosomal mosaicism, confined placental mosaicism is found in approximately 20% of the POCs, which is higher than the reported rate of 1–2% seen in viable pregnancies in chorionic villus sampling^53,59–61^. Additionally, when we compared the nature and prevalence of genomic aberrations along the gestational weeks of the first trimester, we observed that, on average, there may be a higher level of mosaicism in earlier POCs (Supplementary Tables 2 and 5). For instance, for mitotic aberrations in extra-embryonic mesoderm (*n* = 14), the average level of mosaicism in POCs of gestational weeks 4–7 (*n* = 7) was 67.9% ± 26.4 s.d., whereas, in POCs of gestational weeks 8–13 (*n* = 7), the average level of mosaicism was 48.9% ± 30.8 s.d. (*P* = 0.33, two-sided Mann–Whitney *U*-test; Supplementary Table 5). Our cohort is underpowered to draw firm conclusions regarding the comparison of different gestational ages. To reach a sufficient power of 0.8, a minimum sample size of 86—that is, 43 POCs per time interval—would be required (Methods). In addition, a complete allelic architecture of pregnancy loss in the second and third trimesters in future studies will reveal a more detailed etiology of pregnancy losses that are caused by pre-zygotic or post-zygotic chromosomal alterations.

This study has practical implications, contributing to the emerging studies using NIPT in RPL^62^. Specifically, in the case of detected copy number changes after NIPT, genome haplarithmisis can distinguish between meiotic and mitotic errors. NIPT with confined placental mosaicism can lead to false-positive test results. These findings require fetal invasive genetic testing that could potentially be avoided by accurate detection of the segregational origin of the mutation as well as linking low abundant cell-free DNA (cfDNA) to the fetal or placental lineage, which is required to exclude the presence of aberrant cells in the fetus. Aberrations of meiotic origin, if occurring via rescue events into mosaic blastocysts, are likely affecting both fetal and placental lineages and predisposing for spontaneous miscarriage, whereas mitotic aberrations may be specific to the placenta due to confined placental mosaicism^10^ and are compatible with healthy pregnancies. This allows differentiating low-risk pregnancy aneuploidies with aneuploidies of mitotic origin where the fetus may not be affected from higher-risk pregnancies with severe clinical consequences where the fetus is likely affected, as meiotic aberrations are present in both the fetal and placental lineages. Additionally, epigenetic studies of cfDNA have suggested that fetal tissues other than the placenta-derived trophoblasts may also contribute to the cfDNA mixture of maternal blood^63^. This makes it possible to differentiate confined placental mosaicism, which is apparently safe for pregnancy, from a situation where (mosaic) aberration is present in both placental tissue and the fetus, with a higher risk for pregnancy loss. This underlines the importance of developing technologies that can reliably identify placental and fetal origin when an aberration is found in early pregnancy. Moreover, haplotyping-based NIPT methods enable a generic approach for detection of monogenic disorders^64^.

Taken together, the detection of the segregational origin of chromosomal aberrations is of paramount importance for prognosing the successful completion of pregnancy. Although embryos with mosaic abnormalities can lead to the birth of healthy babies^65^, the meiotic or mitotic origin of mosaicism has not always been determined in these successful cases. For instance, as in PGT of IVF embryos, the DNA sample is derived from a single trophectoderm biopsy, and the true extent of mosaicism in all embryo compartments, including the inner cell mass, cannot be determined. However, the determination of the segregational origin of mosaic aberrations would help to avoid transferring meiotic (mosaic) IVF embryos, which confer a high risk for inner cell mass aberrations and later pregnancy loss. In contrast, we suggest that the rules for selecting mitotic mosaic embryos for uterine transfer could be more relaxed.

In conclusion, our study shows that as many as two-thirds of all pregnancy losses may be due to fetal chromosomal abnormalities. Furthermore, the ability to accurately determine the segregational and lineage origins of fetal genomic aberrations may enhance the efficacy of human natural and assisted conception and, thereby, improve reproductive genetic care in general.

## Methods

### Ethical approval

Embryonic tissues and parental blood samples were obtained from the Biobank of Populations of Northern Eurasia, Research Institute of Medical Genetics, Tomsk National Research Medical Center. All couples signed an appropriate informed consent for the transfer of their samples to the biobank for scientific research. This study was approved by the local ethics committee of the Research Institute of Medical Genetics, Tomsk National Research Medical Center, of the Russian Academy of Sciences (protocol no. 15 February 2021). Permission was given for the retrospective analysis of the anonymized biological samples of the biobank.

### Ultrasound diagnosis of early pregnancy loss

The ultrasonography features of early pregnancy loss considered in this study were no cardiac activity or empty gestational sac with a diameter ≥25 mm, crown–rump lengths (CRLs) ≥7 mm for embryos with no cardiac activity, the absence of an embryo and its cardiac activity 14 d after the detection of a gestational sac without a yolk sac and the absence of an embryo and its cardiac activity 11 d after the detection of a gestational sac with a yolk sac^66^. Anembryonic pregnancy (AP) was diagnosed in the absence of an embryo in the gestational sac for a period of more than 7 weeks; in addition, ultrasound criteria for AP were a gestational sac ≥13 mm without a yolk sac or ≥18 mm without an embryo. Missed abortion (МА) was diagnosed for embryos with CRL ≥7 mm without cardiac activity or no cardiac activity upon the initial scan and post-7-days scan for embryos with CRL <7 mm. Spontaneous abortion (SA) was diagnosed as a spontaneously terminated pregnancy without ultrasound examination. The most frequent clinical forms of early pregnancy loss in our study were MAs, followed by APs and SAs (Table 1). After ultrasonography diagnosis, women were admitted to gynecological clinics for curettage or medication abortion. Extra-embryonic tissues or fragmented gestational sacs were collected in sterile saline and immediately transferred to the Laboratory of Cytogenetics, Research Institute of Medical Genetics, Tomsk National Research Medical Center, for cytogenetic analysis and cryopreservation.

### Sampling of POCs

The POCs, usually represented by fragments of the gestational sac, were delivered to the laboratory in sterile saline, thoroughly washed and separated from decidual tissues and blood clots under an inverted microscope. Part of each tissue sample was used for cell culture, and the remaining tissue sample was stored at −70 °C for DNA extraction. Traditionally, cytogenetic studies of POCs have used one of two methods to determine the karyotype^53^. The first involves long-term culture of extra-embryonic tissues; most often, cells that are cultured are derived from the stroma of the chorionic villi^47^. The second approach exploits spontaneously and rapidly dividing cells of the cytotrophoblast to obtain direct chromosome preparations without culturing^67,68^. Usually, the results of both techniques are similar. However, discordant results can be obtained in some cases due to tissue-specific placental mosaicism^13^. Therefore, when large fragments of fetal sac were present, the internal mesodermal layer of extra-embryonic membrane was used for cell culture; otherwise, both extra-embryonic mesoderm and chorionic villi were used (see the ‘Chorionic villi and extra-embryonic mesoderm dissection of the POCs and DNA extraction’ subsection). The tissues were chopped with scissors into small fragments, and long-term cultures were set up in 25-cm^2^ flasks with 5 ml of DMEM/F12 (1:1) medium (Gibco) supplemented with 20% FBS (HyClone), 1% MEM NEAA solution (Gibco) and 1% penicillin–streptomycin (Gibco). Tissues were incubated at 37 °C with once-weekly medium renewal. Extra-embryonic fibroblasts were cultivated until sufficient mitotic cells for cytogenetic analysis were obtained. Demecolcine (Sigma-Aldrich) was added 4 h before chromosome harvesting, and the samples were processed using standard techniques of hypotonic treatment with 0.55% sodium citrate and cell fixation with a 1:3 mixture of acetic acid:methanol. In some cases, direct preparations of the chorionic villi were used^69^. Slide preparations and GTG banding were performed by standard protocols in accordance with guidelines^70^. Subsequently, frozen tissues were used for DNA extraction and preparation of cell suspensions for interphase FISH.

### Chorionic villi and extra-embryonic mesoderm dissection of the POCs and DNA extraction

Genomic DNA was extracted from blood samples of the parents and from two distinct locations in the POC: chorionic villi and extra-embryonic mesoderm. The dissection of chorionic villi and extra-embryonic mesoderm by an experienced pathologist is possible from the fourth week of gestation onwards, whereas, from the sixth week of gestation, the separation of extra-embryonic mesoderm and chorionic villi almost always succeeds (the mean gestational week in this study is 7.5 ± 1.7 s.d.). Specifically, after thawing, chorionic villi were carefully scraped off under an Axiovert 200 inverted microscope (Carl Zeiss) from extra-embryonic membranes based on their morphology, and DNA was extracted separately from these tissues of each sample. The main limitation for accurate dissection of chorionic villi and extra-embryonic mesoderm, however, is the way that POCs are acquired after pregnancy termination. This is because different methods after pregnancy termination are used in medical practice, including curettage, vacuum aspiration or using specific drugs. Genomic DNA was extracted using a standard phenol‒chloroform extraction method that allows for the isolation of up to 900 ng of DNA from the tissues. To isolate DNA from tissues, a small fragment of tissue (200–300 mg) was taken. Then, samples of chorionic villi or extra-embryonic mesoderm were placed in Eppendorf tubes, and 467 µl of buffer (1 ml of 1 M Tris-HCl, pH 7.4; 2 ml of 0.5 M EDTA; 200 µl of 5 M NaCI; 96.8 ml of water) was added to them, in addition to 25 µl of 20% SDS and 7.5 µl of proteinase K (10 mg ml^−1^). The samples were incubated for 16 h at 37 °C. Then, 550 μl of phenol was added, mixed gently and centrifuged for 3 min at 12,000 r.p.m. and room temperature. Next, 300 μl of phenol and 300 μl of a mixture (chloroform:isoamyl alcohol in a ratio of 24:1) were added to the supernatant liquid, mixed and centrifuged under the same conditions. Afterwards, 550 μl of the mixture (chloroform:isoamyl alcohol) was added to the supernatant, mixed and centrifuged. The supernatant liquid was taken again; 30 μl of 10 M sodium acetate and 660 μl of ethanol were added; and the tube was inverted until DNA was visualized. The solution was removed; 100 μl of 70% ethanol was added to the DNA; the DNA was washed and centrifuged at 12,000 r.p.m. for 3 min; the alcohol was removed; and the precipitate was dried at 37 °C. DNA was dissolved in 100 µl of Tris/EDTA buffer. Likewise, genomic DNA was extracted from the peripheral blood of parents using the standard phenol‒chloroform method.

### Conventional CGH and interphase FISH

Conventional CGH and interphase FISH with centromere enumeration probes were performed as described previously^60,71^ for POC samples where traditional cytogenetic analysis failed. For interphase FISH, two tissues were mechanically separated, and the yield of chorionic cytotrophoblast cells was increased by maceration of chorionic villi under an Axiovert 200 inverted microscope (Carl Zeiss) and treatment with 70% acetic acid for 3–5 min, followed by three washes of the obtained cell suspension with PBS according to a modified protocol^67^. The extra-embryonic mesoderm cells were obtained by digesting the extra-embryonic membrane (Fig. 3a) with 125 U ml^−1^ collagenase type I (Sigma-Aldrich) for 30–60 min at 37 °C (ref. ^60^). Cell suspensions were fixed and stored in 3:1 methanol:acetic acid at −20 °C. For confirmation studies, interphase FISH was performed separately for thawed chorionic villi and extra-embryonic mesoderm cells using centromere-specific DNA probes for chromosomes 2, 15, X and Y as well as subtelomeric DNA probes for chromosome 16 (16p, 16q). From 100 to 400 interphase nuclei were scored for each sample using an Axio Imager Z2 microscope (Carl Zeiss) with Metafer and ISIS software (MetaSystems).

### Selection criteria for the participating patients

From 1987 to 2021, a total of 1,745 spontaneous pregnancy losses were analyzed using karyotyping. The karyotypes of fetal tissue were determined by conventional metaphase analysis (*n* = 1,745 and, on average, 10 metaphases) or additional testing by CGH and FISH. All samples with abnormal karyotypes were excluded from downstream haplarithmisis. Of the karyotypically normal cases, 111 families (114 POCs) were randomly selected for SNP haplotyping, given that fetal (extra-embryonic mesoderm and chorionic villi) and parental blood samples were available and that no genetic predisposition for pregnancy loss had been identified in the couple. Twenty families were excluded due to the DNA of one or more family members being of insufficient quantity or quality, causing low SNP call rates or due to one or both parents not being the biological parent, making haplotyping analysis impossible (Supplementary Table 6). Ninety-one families (94 POCs) were successfully analyzed by haplarithmisis. Of those, 42 were categorized as SPL (loss of one pregnancy) and 49 as RPL (loss of two or more consecutive pregnancies).

### Whole-genome SNP genotyping

SNP genotyping was performed on genomic DNA isolates using Illumina Infinium Global-Screening Array-24 version 2.0 and version 3.0 BeadChip Kit (Illumina; Gene Expression Omnibus (GEO) code GLP28939), which contains approximately 665,000 SNP markers with a mean probe spacing of ~4.4 kb and a median probe spacing of ~2.3 kb. Genotype calls, SNP BAF values and logR values of all samples were computed using Illumina GenomeStudio software. Illumina genotyping was performed at the Core Facility of Genomics, Institute of Genomics, University of Tartu, Estonia.

### Genome haplarithmisis

Haplarithmisis is a conceptual workflow that enables simultaneous genome-wide haplotyping and copy number typing using genotyping information from offspring and parents^7^. This originally allowed tracing the inheritance of linked disease variants. Previously, we demonstrated that, using Illumina’s Global-Screening Array-24, haplarithmisis can detect low-grade mosaicism (>10%), subchromosomal CNVs (>100 kb), the parental and segregational origin of aberrations and maternal cell contamination in placenta^10^.

Specifically, in the present study, the parental genotypes were phased by using the chorionic villi genotype of the POC as a seed for phasing. Subsequently, the BAF values—that is, continuous genotype values (Fig. 2a)—of the extra-embryonic mesoderm and chorionic villi are deduced to parental haplarithms (Fig. 2b). Specifically, (1) informative SNP loci are defined when one parent is heterozygous and the other parent homozygous; and (2) these SNP loci are further categorized into maternal and paternal categories. A paternal category is all the SNP loci that have a heterozygous SNP from the father and a homozygous SNP from the mother. Similarly, a maternal category is all the SNP loci that have heterozygous SNP from the mother and homozygous SNP from the father. Subsequently, (3) a subcategorization is made based on (phased) parental SNP genotype combinations into paternal subcategories P1 and P2 (shown in Fig. 2b) and maternal subcategories M1 and M2 (not shown in Fig. 2b). (4) This results in specific P1 and P2 in paternal haplarithm, depending on homolog inheritance—for example, if homolog 1 (H1) is inherited from the father (and either H1 or H2 from the mother), P1 BAFs are either 0 or 1 (corresponding to homozygous AA and BB genotypes, respectively), and P2 BAFs are 0.5 (corresponding to heterozygous AB genotype). In contrast, when H2 is inherited from the father (and either H1 or H2 from the mother), P1 BAFs are 0.5 (corresponding to heterozygous AB genotype), and P2 BAFs are either 0 or 1 (corresponding to homozygous AA and BB genotypes, respectively). M1 and M2 maternal subcategories are computed in a similar fashion. (5) BAF values are mirrored around the 0.5 axis for SNPs where either parent has a heterozygous SNP call BA after phasing. Thus, if H1 was inherited from the father (and either H1 or H2 from the mother), all P1 BAF values will now have a value of 0, and P2 BAF values will continue to have a value of 0.5. In contrast, if H2 was inherited from the father (and either H1 or H2 from the mother), all P1 BAF values will continue to have a value of 0.5, and all P2 BAF values will now have a value of 1. The same computation applies to P2, M1 and M2 BAFs. Mirroring of these specific values for P1 and P2 allows detection of homologous recombination between the paternal H1 and H2—the same for M1 and M2 maternal subcategories. (6) Per subcategory, consecutive parental BAF values are segmented by piecewise constant fitting (PCF; segmentation parameter gamma set to 14 in this study). (7) The paternal (P1 and P2) and maternal (M1 and M2) segments are visualized into two separate haplarithm plots. Segmented paternal P1 and P2 BAFs are depicted in blue and red, respectively, as are maternal M1 and M2 BAFs in blue and red, respectively. (8) Paternal and maternal haplarithms reveal haplotypes (imbalances) and their parental and segregational origin.

As described previously in detail^7^, haplarithmisis has two features for detection of degree of mosaicism (Fig. 2c) and segregational origin (Fig. 2d). First, the parity within each parental haplarithm where the length of P1 and P2 segments should approximately correspond to breakpoints of homolog recombination (similarly for M1 and M2 segments). Second, the reciprocity between parental profiles where the difference between P1 and P2 BAFs (d_pat_) in combination with the difference between M1 and M2 BAFs (d_mat_) is characteristic for specific abnormalities, and their specific pattern correlates to segregational origin. For example, if d_pat_ has a value of 0.67 and d_mat_ has a value of 0.33, and the logR value for this chromosome is raised above 0 (~0.58) as compared to the other chromosomes, this is indicative of a trisomy where the maternal chromosome has an abnormal number of two copies—that is, 1_paternal_:2_maternal_ allelic ratio. Subsequently, segregational origin can be determined by the position of the M1 and M2 values around the centromere. These values around the centromere change depending on whether the centromere contains three different homologs (H1 maternal, H2 maternal and H1 paternal, indicating meiosis I) or two different homologs (H1 maternal and H1 paternal, indicating meiosis II or mitosis). To distinguish between meiosis II and mitotic errors, the M1 and M2 values effected by a recombination event are used. This depends on whether there are three different homologs (H1 maternal, H2 maternal and H1 paternal, indicating meiosis II) or two different homologs (H1 maternal and H1 paternal, indicating mitosis) at the recombination event. To determine the degree of mosaicism, the genomic coordinates at the logR distortion from the expected value—that is, logR = 0—were used to extract the BAFs and segmented P1, P2 or M1, M2 BAFs of the location of interest. BAF values were then compared to the reference dataset for calculation of level of mosaicism, as described previously^19^.

Haplarithmisis was applied to each quartet DNA sample to delineate the allelic architecture of the fetal tissues. Chorionic villi tissue was used as a reference to phase parental genotypes. Parental haplarithms were used to infer the DNA copy number state, parent of origin and level of mosaicism of fetal tissues. In total, 450 DNA samples were analyzed. Levels of mosaicism were calculated as previously described^19^. Parent-of-origin haplotyping allows for the detection of maternal DNA contribution in fetal tissues, as previously described^10^. In one DNA sample, complete maternal contamination was detected by genome haplarithmisis and validated by quantitative fluorescence polymerase chain reaction (QF−PCR). This DNA sample was excluded from the study.

### Classification of (segmental) chromosomal abnormalities

Haplarithms of analyzed tissues were classified based on several factors: types of aberrations detected, size of aberrations (genome-wide/chromosomal/segmental), placental or embryonic origin based on tissue biopsy, parental (paternal, maternal) and segregational (mitotic, meiosis I, meiosis II) origin and level (%) of mosaicism. Levels of mosaicism were calculated based on BAF values as previously described^19^. Extrapolation of the total abnormalities was calculated using the formula below:

$$\left(\left({\rm{AK}}+\left(\frac{{\rm{NK}}{\rm{\times }}{\rm{AH}}}{{\rm{TH}}}\right)\right){\rm{\times }}100\right)/{\rm{TK}}=67.8 \%$$

where AK is the number of abnormal cases by conventional karyotyping; NK is the number of normal cases by conventional karyotyping; AH is the number of abnormal cases by genome haplarithmisis; TH is the total number of cases by genome haplarithmisis; and TK is the total number of cases by conventional karyotyping.

### Other statistical analyses

Comparisons between conventional karyotyping (*n* = 1,745) and genome haplarithmisis (*n* = 91 families, 94 POCs) concerning parental and gestational age were performed by two-sided Welch’s *t*-test. For dichotomous outcomes, the chi-squared test was applied to analyze parental and segregational origin outcomes of SPL and RPL. Due to low sample size (non-parametric) and paired samples (extra-embryonic mesoderm and chorionic villi from a single fetus), the Wilcoxon signed-rank test was applied to assess the difference in mosaicism degree between extra-embryonic mesoderm and chorionic villi. To calculate the required sample size for the Wilcoxon signed-rank test for matched pairs to test the difference in mosaicism between extra-embryonic mesoderm and chorionic villi, we assumed a normal parent distribution, a mean percentage of 43% mosaicism in the chorionic villi group, an s.d. of 30 in both groups and a correlation between the groups of 0.5. A total sample size of 35 participants was required to test a 15% difference between the extra-embryonic mesoderm and chorionic villi groups, performing a two-sided test using an alpha of 0.05 and a power (1-β) of 0.80. The sample size calculation was performed using G*Power 3.1.9.7. In addition, a power calculation was performed for mosaicism dynamics across gestational age bins. A total sample size of 86 samples was required to test a 15% difference between week bins 4–7 (*n* = 7) and 8–13 (*n* = 7) for extra-embryonic mesoderm tissues containing aberrations of mitotic origin, performing a two-sided test using an alpha of 0.05 and a power of (1-β) of 0.80, indicating that our study is underpowered concerning mosaicism dynamics across gestational age, with a power of (1-β) of 0.18 due to low sample size (Supplementary Tables 2 and 5). To assess the relation between mosaicism and gestational age week bins (4–7 and 8–13), two-sided Mann–Whitney *U*-test was performed (Supplementary Table 5).

### Methylation profiling: sample selection and processing

For the methylome analysis, seven POCs (13 DNA samples) were randomly selected based on the following inclusion criteria: (1) availability of sufficient extracted DNA; (2) female sex (to eliminate potential sex differences from the analysis); and (3) absence of chromosomal abnormalities according to haplarithmisis. Extracted DNA from both chorionic villi and extra-embryonic mesoderm tissues was processed as follows: 300 ng of DNA from each sample was bisulfite converted using the EZ DNA Methylation Kit (Zymo Research) according to the manufacturer’s instructions and analyzed using the Illumina Infinium MethylationEPIC version 1.0 BeadChip Kit (Illumina; GEO code: GLP21145), thereby allowing us to examine DNA methylation at more than 850,000 CpG sites across the human genome. The Illumina methylation array was performed at the Core Facility of Genomics, Institute of Genomics, University of Tartu, Estonia.

### Methylation data processing and analysis

Data pre-processing was carried out using the RnBeads R package as previously described^72,73^. In brief, the data were normalized with subset-quantile within array normalization (SWAN)^74^, and poor-quality sites and samples were removed based on the Greedycut algorithm (detection *P* value threshold: 0.05). The following additional sites were removed: (1) sites on the sex chromosomes, (2) sites near SNPs, (3) sites with missing values in more than 10% of samples and (4) sites not in a CpG context. Additional sample quality control—namely, sex prediction and SNP probe analysis—was carried out using the sEst^75^ package and RnBeads, respectively. Methylation beta values, representing the methylated signal intensity divided by the sum of the methylated and unmethylated signal intensity, were used for all analyses. Cellular deconvolution of the samples was performed using the reference-based Houseman algorithm^76^ on data pre-processed with the recommended preprocessNoob^77^ method and implemented using the minfi^78^ package. For this, the reference site and cell type data for stromal, Hofbauer, endothelial, trophoblast, syncytiotrophoblast and nucleated red blood cells (NRBCs) provided by Yuan et al.^79^ were used. Welch’s *t*-tests were applied for statistical comparison of the cellular proportions predicted in extra-embryonic mesoderm and chorionic villi samples. All high-quality samples and CpG sites were used to conduct a PCA in which beta values were centered but not scaled. The significance of the associations between the principal components (PCs) and sample features was tested as follows: (1) permutation tests (with 10,000 permutations) for continuous numerical variables (gestational age, stromal cells, Hofbauer cells, endothelial cells, NRBCs and syncytiotrophoblast cells) and (2) two-sided Wilcoxon rank tests for binary categorical variables (tissue type).

### RT‒qPCR validation of PL2074 monosomy and uniparental disomy

The haplarithm of PL2074 shows 50% mosaic monosomy 7 in the extra-embryonic mesoderm and 30% uniparental disomy (UPD) in the chorionic villi. To validate these findings, RT‒qPCR was performed with a diploid control, a hemizygous deletion case, two times diluted DNA from the hemizygous deletion case and extra-embryonic mesoderm and chorionic villi from PL2074. Primers for exon 16 of the *WDR60* gene, which is located at 7q36.3, were used. The reference gene was *HEXB*, which encodes the β subunit of hexosaminidase and is located at 5q13. Reference genomic DNA was obtained from the peripheral blood lymphocytes of a healthy donor. The results confirmed the diploid genome in chorionic villi and the mosaic monosomy in extra-embryonic mesoderm (Extended Data Fig. 6a,b). The combination of monosomy and UPD may be indicative of monosomy rescue where the single homolog is duplicated (Extended Data Fig. 6c).

### RT‒qPCR validation of PL1758 and segregational origin of aberrations

The haplarithm of PL1758 shows a genome-wide triploidy of maternal error origin, with mosaic tetrasomy of Chr 2 and Chr 7. The segregational origin appears to be mitotic (Chrs 1, 4 and 12) or meiotic II (Chrs 3, 5, 6, 8, 9, 10, 11, 13, 14, 15, 16, 17, 18, 19, 20, 21 and 22). To validate whether haplotyping with chorionic villi as a reference for extra-embryonic mesoderm would yield accurate parent-of-origin results, additional haplotyping with two different siblings and grandparents from the maternal side as references was performed. The results from PL1758 confirm the segregational origin of the aberrations, even when chorionic villi is used as a reference (Extended Data Fig. 7). RT‒qPCR with reference DNA from spontaneous pregnancy loss with 69,XXX karyotype and primers for exon 12 of the *MBD5* gene (2q23.1), exon 12 of the *ASXL2* gene (2p23.3) and exon 1 of the *CHCHD2* gene (7p11.2) were used to confirm tetrasomy for Chr 2 and Chr 7 in DNA from chorionic villi of PL1758. The following calculations and formulas were used to determine the fold change between the copy number of the test loci in the pregnancy loss and reference DNA: average value for three C_T_; logQT test primer = (C_T_ test DNA − C_T_ reference DNA) / slope test primer; (logQT test primer − log QT control primer); fold change = 10^logQT test primer −^ ^logQT control primer^. Fold change values were used to build a chart (Extended Data Fig. 7b–d). Usually, fold change for reference DNA is 1—namely, there are two copies of the product for reference DNA. Variation from 0.8 to 1.2 in test DNA corresponds to two copies of DNA. Variation from 0.3 or lower to 0.7 (average 0.5–1 copy against two copies) indicates deletion, and variation from 1.3 to 1.7 (average 1.5–3 copies against two copies) indicates duplication. If both reference and test DNA are triploid, and there is a tetrasomy for some chromosome in the test DNA, then the fold change should be approximately 1.33 (that is, four copies against three).

### Short tandem repeat analysis

To confirm relationships and exclude maternal cell contamination, analysis of short tandem repeats (STRs) was carried out using the COrDIS EXPERT 26 Kit for DNA identification of 26 STR markers (Gordiz). The analysis included identification of 26 loci: *AMEL*, *SRY*, *D3S1358*, *TH01*, *D12S391*, *D5S818*, *TPOX*, *Yindel*, *D2S441*, D7S820*, D13S317*, *FGA*, *D22S1045*, *D18S51*, *D16S539*, *D8S1179*, *CSF1PO*, *D6S1043*, *VWA*, *D21S11*, *SE33*, *D10S1248*, *D1S1656*, *D19S433*, *D2S1338* and *DYS391*. PCR products were fractionated using an ABI PRISM 3130 HID capillary electrophoresis system (Applied Biosystems). Fragment length was determined using internal length standards (Size Standard GeneScan 550) and GeneMapper 4.1 software.

### Comparative genomic hybridization array for aneuploidy detection

Chorionic villi DNA samples from SAs and control male and female DNA samples were labeled by a SureTag Labeling Kit (G9502A, Agilent) and purified by SureTag Purification Columns (5190-7730, Agilent). Labeled DNA samples were hybridized using the GenetiSure Pre-Screen Complete Kit (8 × 60K) (G5963A, Agilent) at 67 °C for 24 h, according to the manufacturer’s protocol. Microarray images were obtained using a SureScan Microarray Scanner (Agilent) and analyzed by Agilent Feature Extraction (version 12.2.0.7) and CytoGenomics software (version 5.2).

### Reporting summary

Further information on research design is available in the Nature Portfolio Reporting Summary linked to this article.

## Online content

Any methods, additional references, Nature Portfolio reporting summaries, source data, extended data, supplementary information, acknowledgements, peer review information; details of author contributions and competing interests; and statements of data and code availability are available at 10.1038/s41591-023-02645-5.

### Supplementary information

Supplementary Tables 1–6.

Reporting Summary

### Source data

Source data for Fig. 1c,d.

Source data for Supplementary Table 2.

## Data Availability

All SNP array and methylation data generated in this study were deposited in the National Center for Biotechnology Informationʼs Gene Expression Omnibus (http://www.ncbi.nlm.nih.gov/geo/) under accession code GSE228151. Source data are provided with this paper.

## Code availability

Custom code is available on GitHub: https://github.com/CellularGenomicMedicine/Pregnancy_loss_study.
