# Supplementary material for: Prevalence of chromosomal alterations in first-trimester spontaneous pregnancy loss
Source: Nat Med. 2023 Nov 23;29(12):3233–42. doi: 10.1038/s41591-023-02645-5 (PMC10719097; doi:10.1038/s41591-023-02645-5)
Supplement: Supplementary file 1 — Supplementary Tables 1–6. [file 41591_2023_2645_MOESM1_ESM.pdf]

nature medicine

Article

https://doi.org/10.1038/s41591-023-02645-5

Prevalence of chromosomal alterations in first-trimester spontaneous pregnancy loss

In the format provided by the authors and unedited

Supplementary Table 1 | Logistic regression analysis of parental, gestational age and abnormality status.

Maternal, paternal and gestational age on genomic status of tissue samples (normal, abnormal)
Coefficients and confidence intervals

	Estimate	Std. Error	Z value	Pr(>|z|)	2.5 %	97.5 %
(Intercept)	-0.44421	0.40332	-1.101	0.271	0,28993311	1,41106131
Paternal age	0.01244	0.01346	0.924	0.356	0,98624736	1,0397907
Maternal age	0.00842	0.01477	0.570	0.569	0,9796628	1,03814665
Gestational age	0.01627	0.02902	0.561	0.575	0,96049016	1,07655417

Maternal and paternal age on genomic status of tissue samples (normal, abnormal)
Coefficients and confidence intervals

	Estimate	Std. Error	Z value	Pr(>|z|)	2.5 %	97.5 %
(Intercept)	-0.59236	0.28226	-2.099	0.0358	0,3173412	0,96013088
Paternal age	0.01410	0.01191	1.184	0.2365	0,99087323	1,03829935
Maternal age	0.01381	0.01277	1.081	0.2795	0,98885307	1,03967305

Paternal age on genomic status of tissue samples (normal, abnormal)
Coefficients and confidence intervals

	Estimate	Std. Error	Z value	Pr(>|z|)	2.5 %	97.5 %
(Intercept)	-0.478889	0.262114	-1.827	0.06770	0,36967328	1,03356382
Paternal age	0.023349	0.008339	2.800	**0.00511**	1,00711936	1,04061187

Maternal age on genomic status of tissue samples (normal, abnormal)
Coefficients and confidence intervals

	Estimate	Std. Error	Z value	Pr(>|z|)	2.5 %	97.5 %
(Intercept)	-0.856824	0.229770	-3.729	**0.000192**	0,27010982	0,66507459
Maternal age	0.031344	0.007919	3.958	**7. 55e-05**	1,01600643	1,04805353

Supplementary Table 2 | Aberrations detected in POCs after PL by genome haplarithmisis.

Sample	Gestational age by ultrasound (week)	Tissue	Aberration	Chr.	Size (Mb)	Mosaicism (%)
PL2650	4.5	CV	Trisomy	16	88.7	90
		EM	Trisomy		88.7	97.5
PL140	4.5	CV	Trisomy	16	88.7	87.5
		EM	Trisomy		88.7	85
PL2074	5.5	CV	UPD	7	160.6	30
		EM	Monosomy		160.6	50
PL2682	5.5	CV	Trisomy	19	61.7	35
		EM	Trisomy		61.7	90
PL1618	6.0	CV	Duplication	5	~2.7	20
		EM	Duplication		~2.7	17.5
PL2660	6.0	CV	Trisomy	14	101.2	85
		EM	Trisomy		101.2	87.5
PL2678	6.0	CV	Trisomy	16	88.7	97.5
		EM	Trisomy		88.7	97.5
PL1400	6.1	CV	Trisomy	16	88.7	85
		EM	Trisomy		88.7	87.5
PL1783	6.1	CV	Trisomy	2	242.7	97.5
		EM	Trisomy		242.7	85
PL2733	6.2	CV	Trisomy	16	88.7	90
		EM	Trisomy		88.7	90
PL2726	6.4	CV	Normal	GW	-	0
		EM	Trisomy	13	113.6	80
		CV	UPD	1	248.4	15
		EM	UPD		248.4	65
PL2727	6.5	CV	Trisomy	9	150.6	100
		EM	Trisomy		150.6	97.5
PL2653	6.7	CV	Trisomy	2	242.7	100
		EM	Trisomy		242.7	100
PL2087	7.0	CV	Trisomy	15	99.8	90
		EM	Trisomy		99.8	85
PL1744	7.0	CV	Trisomy	22	51.3	87.5
		EM	Trisomy		51.3	87.5
PL2621	7.4	CV	Trisomy	7	160.6	97.5
		EM	Trisomy		160.6	100
PL1595	7.5	CV	Trisomy	18	80.5	87.5
		EM	Trisomy		80.5	87.5
PL2016	7.5	CV	Polyploidy	GW	-	NA
		EM	Polyploidy		-	NA
PL1359	8.0	CV	Trisomy	6	172.1	87.5
		EM	Trisomy		172.1	87.5
PL2019	8.0	CV	Deletion	1	~44.7	30
		EM	Deletion		~44.7	40
		CV	Duplication	4	~18.3	5
		EM	Duplication		~18.3	32.5
PL401	8.0	CV	Monosomy	11	135.1	10
		EM	Monosomy		135.1	10
PL2728	8.1	CV	Trisomy	15	99.8	90
		EM	Trisomy		99.8	90

PL1701	8.4	CV	Trisomy	5	182.0	10
		EM	Trisomy		182.0	37.5
PL2702	8.5	CV	Trisomy	15	99.8	90
		EM	Trisomy		99.8	87.5
PL444	8.5	CV	Trisomy	14	101.2	45
		EM	Trisomy		101.2	72.5
PL1896	8.6	CV	Monosomy	X	154.3	100
		EM	Monosomy		154.3	100
PL2452	8.6	EM	Trisomy	21	45.1	100
		CV	Trisomy		45.1	100
PL2451	9.5	CV	Polyploidy	GW	-	100
		EM	Polyploidy		-	95
PL2701	10.3	CV	Trisomy	15	99.8	95
		EM	Trisomy		99.8	95
PL2137	13.0	CV	Trisomy	7	160.6	30
		EM	Trisomy		160.6	40
PL1758	NA	CV	Tetrasomy	2	242.7	NA
		EM	Tetrasomy		242.7	NA
		CV	Tetrasomy	7	160.6	45
		EM	Tetrasomy		160.6	40
		CV	Polyploidy	GW	-	100
		EM	Polyploidy		-	90
PL2223	NA	CV	Monosomy	X	154.3	35
		EM	Normal	GW	-	0
PL245	NA	CV	Deletion	5	~16.7	80
		EM	Deletion		~16.7	80
		CV	Duplication	7	~43.2	35
		EM	Duplication		~43.2	45

Supplementary Table 3 | Chromosomal microarray studies of PLs with >1000 samples, without previous karyotyping.

№	Study	Clinical specimen	Microarray technology	Total cases (#)	Cases with chromosomal abnormalities (#)	Prevalence (%)
1	Levy et al., 2014 (PMID: 25004334)	Miscarriages (<20 weeks of gestation)	Illumina CytoSNP-12	1861	1118	60.1
2	Zhou et al., 2016 (PMID: 27020032)	POC from PL	Agilent 60K aCGH	1235	507	41.1
3	Chen et al., 2017 (PMID: 28247551)	POC from PL	Version 7.6 Oligo, Baylor, WGS	2186	975	44.6
4	Peng and Yuan, 2018 (PMID: 30369481)	POC from PL	Affymetrix CytoScan 750K	2505	959	38.3
5	Li et al., 2020 (PMID: 32626971)	Miscarriages	Agilent aCGH, 60K, lc-NGS	1401	693	49.5
6	Wang et al., 2021 (PMID: 33069876)	Miscarriages (<13 weeks of gestation)	Affymetrix CytoScan 750K, QF-PCR, HLPA	1042	698	67.0
7	Finley et al., 2022 (PMID: 35523710)	POC (fresh/FFPE) from PL	Illumina CytoSNP-12	24900	13909	55.9
8	Sahoo et al., 2017 (PMID: 35523710)	RPL	CMA or SNP-array (3520 clones, BAC-clone-based array-CGH (CombiMatrix), 180334 oligonucleotide probes oligonucleotide array (Agilent) or CytoSNP-850 K array (Illumina))	7396	3975	53.7
**Total**				**42526**	**22834**	**53.7**

Supplementary Table 4 | Chromosomal microarray studies of PLs, where karyotyping was performed previously by conventional karyotyping techniques.

№	Study	Clinical specimens	Methods	Total cases with normal karyotype	Additionally detected chromosomal abnormalities	%
1	Shimokawa et al., 2006 (PMID: 16906550)	PLs with normal karyotype	BAC Array – 1 Mb System, Spectral Genomics, Houston. TX. USA	20	2	10.0
2	Zhang et al., 2009 (PMID: 19215247)	First-trimester PLs	oligonucleotide-based array-CGH with a 244K chip (Agilent Technologies))	58	13	22.4
3	Warren et al., 2009 (PMID: 20168112)	Fetal loss at 10–20 weeks	Spectral 2600 whole genome BAC array (PerkinElmer) and Agilent 244 K oligonucleotide array to confirm it)	30	4	13.3
4	Rajcan-Separovic et al., 2010 (PMID: 20847186)	RPL	Agilent 105 K oligonucleotides array-CGH	26	11	42.3
5	Kooper et al., 2014 (PMID: 24428858)	Intrauterine fetal death	Affymetrix GeneChip 250 k (NspI) SNP-array) for 71 samples and CytoScan HD, 2.6 M for 97 samples	167	31	18.6
6	Bug et al., 2014 (PMID: 25013457)	POCs	CGH + SNP8×60K microarrays (BlueGnome Ltd., Cambridge, UK) (customized)	34	4	11.8
**Total**				**335**	**65**	**19.4**
	Present study	POCs	SNP-array + Genome haplarithmisis	94	33	35.1
**Total**				**429**	**98**	**22.8**

Supplementary Table 5 | Degree of mosaicism and gestational age in week bins.

Segregational origin	Tissue	Mosaicism % (mean ± sd) Weeks 4-7	n	Weeks 8-13	n	*P* value*
Mitotic	CV	46.4 ± 38.7	7	38.6 ± 38.2	7	1.00
	EM	67.9 ± 26.4		48.9 ± 30.8		0.33
Meiotic	CV	88.2 ± 18.4	11	84.5 ± 22.8	5	1.00
	EM	92.3 ± 6.30		89.0 ± 10.4		0.77
Mitotic and meiotic	CV	71.9 ± 34.1	18	61.0 ± 39.4	13	0.81
	EM	82.8 ± 20.5		68.3 ± 31.5		0.43

*2-sided Mann Whitney U test

Supplementary Table 6 | Excluded samples.

ID	Group	Maternal age	Paternal age	Gestational age	Total PLs	Exclusion reason	Affected member
PL137	SPL	24	25	NA	1	Bad quality DNA	Father
PL142	SPL	34	NA	NA	1	Bad quality DNA	EM & CV
PL158	SPL	25	24	NA	1	Mix up	Mother & father
PL167	SPL	19	22	NA	1	Bad quality DNA	Mother
PL228	SPL	34	34	NA	1	Bad quality DNA	Mother & father & EM & CV
PL241	SPL	30	43	NA	1	Mix up	Father
PL246	SPL	30	30	NA	1	Bad quality DNA	EM & CV
PL250	RPL	29	29	NA	4	Bad quality DNA	Mother & father & EM & CV
PL278	SPL	33	37	NA	1	Bad quality DNA	Mother
PL379	SPL	22	22	10	1	Bad quality DNA	EM & CV
PL384	RPL	24	25	13	2	Bad quality DNA	EM
PL588	RPL	27	27	10	3	Bad quality DNA	Father
PL714	RPL	23	25	6	3	Bad quality DNA	Mother
PL822	SPL	31	26	7	1	Mix up	Mother & father
PL915	SPL	25	33	6	1	Bad quality DNA	EM
PL920	SPL	28	33	5,5	1	Bad quality DNA	Mother
PL1362	RPL	24	23	6	2	Bad quality DNA	EM & CV
PL2178	RPL	25	31	8	3	Bad quality DNA	Mother
PL2730	RPL	30	28	7,4	3	Bad quality DNA	CV
PL2734	RPL	43	37	8,2	7	Mix up	Mother

SPL, sporadic pregnancy loss; RPL, recurrent pregnancy loss; EM, extraembryonic mesoderm; CV, chorionic villi.
