# Supplementary material for: Prevalence of chromosomal alterations in first-trimester spontaneous pregnancy loss
Source: Nat Med. 2023 Nov 23;29(12):3233–42. doi: 10.1038/s41591-023-02645-5 (PMC10719097; doi:10.1038/s41591-023-02645-5)
Supplement: Supplementary file 2 — Reporting Summary [file 41591_2023_2645_MOESM2_ESM.pdf]

nature portfolio

Corresponding author(s): Masoud Zamani Esteki

Last updated by author(s): 25-05-2023

Reporting Summary

Nature Portfolio wishes to improve the reproducibility of the work that we publish. This form provides structure for consistency and transparency in reporting. For further information on Nature Portfolio policies, see our Editorial Policies and the Editorial Policy Checklist.

Statistics

For all statistical analyses, confirm that the following items are present in the figure legend, table legend, main text, or Methods section.

n/a	Confirmed	
☐	☒	The exact sample size (*n*) for each experimental group/condition, given as a discrete number and unit of measurement
☐	☒	A statement on whether measurements were taken from distinct samples or whether the same sample was measured repeatedly
☐	☒	The statistical test(s) used AND whether they are one- or two-sided<br>*Only common tests should be described solely by name; describe more complex techniques in the Methods section.*
☐	☒	A description of all covariates tested
☐	☒	A description of any assumptions or corrections, such as tests of normality and adjustment for multiple comparisons
☐	☒	A full description of the statistical parameters including central tendency (e.g. means) or other basic estimates (e.g. regression coefficient) AND variation (e.g. standard deviation) or associated estimates of uncertainty (e.g. confidence intervals)
☒	☐	For null hypothesis testing, the test statistic (e.g. F, t, r) with confidence intervals, effect sizes, degrees of freedom and P value noted<br>*Give P values as exact values whenever suitable.*
☒	☐	For Bayesian analysis, information on the choice of priors and Markov chain Monte Carlo settings
☒	☐	For hierarchical and complex designs, identification of the appropriate level for tests and full reporting of outcomes
☒	☐	Estimates of effect sizes (e.g. Cohen's *d*, Pearson's *r*), indicating how they were calculated

Our web collection on statistics for biologists contains articles on many of the points above.

Software and code

Policy information about availability of computer code

Data collection	New data was generated for this study. Genome-wide single nucleotide polymorphism profiles in DNAs from maternal and paternal blood, and extraembryonic mesoderm and chorionic villi from the miscarried product of conception of 111 families (114 POCs). SNP genotyping was performed on genomic DNA isolates using Illumina InfiniumTM Global-Screening Array-24 v2.0 and v3.0 BeadChip Kit (Illumina, no. GEO: GLP28939), which contains approximately 665.000 SNP markers with a mean probe spacing of ~4.4 kb and a median probe spacing of ~2.3 kb. Illumina genotyping was performed at the Core Facility of Genomics, Institute of Genomics, University of Tartu, Estonia.
Data analysis	All code will be made available on Github. Genotype calls, SNP B-allele frequency values and logR values of all samples were computed using Illumina GenomeStudio 2.0 software. Haplarithmisis was used to compute parental haplarithms. Raw logR-values were smoothed by using a moving average window of five consecutive SNP probes, wave-corrected for GC% bias by a Lowess fit and normalized to a trimmed mean of normal diploid chromosomes. Normalized logR-values were segmented by piecewise-constant fitting (gamma = 14). Levels of mosaicism were calculated based on BAF values or paternal, maternal haplarithm values.<br>Statistical analysis was performed using RStudio version 2023.03.0+386.

For manuscripts utilizing custom algorithms or software that are central to the research but not yet described in published literature, software must be made available to editors and reviewers. We strongly encourage code deposition in a community repository (e.g. GitHub). See the Nature Portfolio guidelines for submitting code & software for further information.

Data

Policy information about availability of data

All manuscripts must include a data availability statement. This statement should provide the following information, where applicable:

- Accession codes, unique identifiers, or web links for publicly available datasets
- A description of any restrictions on data availability
- For clinical datasets or third party data, please ensure that the statement adheres to our policy

All SNP array data is available on NCBI Gene Expression Omnibus (GEO; http://www.ncbi.nlm.nih.gov/geo/) accession number GEO: GSE228151. Link for access by the reviewers only: https://www.ncbi.nlm.nih.gov/geo/query/acc.cgi?acc=GSE228151 with token number "gfgjigsylrclrst"

Human research participants

Policy information about studies involving human research participants and Sex and Gender in Research.

Reporting on sex and gender	In our retrospective analysis, sex of the miscarried POC DNA was determined by XX/XY SNP genotyping coverage, visualized in haplarithmisis-produced plots. Sex of the couples was obtained from the "Biobank of populations of Northern Eurasia", Research Institute of Medical Genetics, Tomsk National Research Medical Center (https://ckp-rf.ru/catalog/ckp/507500/). The association between abnormality rate in miscarried POCs and paternal and/or maternal age was investigated. In literature, both paternal and maternal age are shown to have an individual effect on the abnormality rate of miscarried POCs.
Population characteristics	In total, 111 couples participated in this study. Age and sex were used in determining the association with abnormality rate in miscarried POCs (see above). Couples (91 families, 94 POCs after exclusion) were divided into sporadic pregnancy loss (42 families and POCs), defined as 1 loss, and recurrent pregnancy loss (49 families and 52 POCs), defined as more or equal to 2 pregnancy losses.
Recruitment	The ultrasonography features of early pregnancy loss considered in this study were no cardiac activity or empty gestational sac with a diameter ≥ 25 mm, crown-rump lengths (CRL) ≥ 7 mm for embryos with no cardiac activity, the absence of an embryo and its cardiac activity 14 days after the detection of a gestational sac without a yolk sac, and the absence of an embryo and its cardiac activity 11 days after the detection of a gestational sac with a yolk sac.<br>The most frequent clinical forms of early pregnancy loss were missed abortions followed by anembryonic pregnancies and spontaneous abortions. After ultrasonography diagnosis, women were admitted to gynecological clinics for curettage or medication abortion. Extraembryonic tissues or fragmented gestational sacs were collected in sterile saline and immediately transferred to the Laboratory of Cytogenetics, Research Institute of Medical Genetics, Tomsk National Research Medical Center (Tomsk, Russia) for cytogenetic analysis and cryopreservation.
Ethics oversight	Embryonic tissues and parental blood samples were obtained from the "Biobank of populations of Northern Eurasia", Research Institute of Medical Genetics, Tomsk National Research Medical Center (https://ckp-rf.ru/catalog/ckp/507500/). All couples signed an appropriate informed consent for the transfer of their samples to the biobank for scientific research. This study was approved by the local Ethics Committee of the Research Institute of Medical Genetics, Tomsk National Research Medical Center of the Russian Academy of Sciences (Protocol #10, February 15, 2021). Permission was given for the retrospective analysis of the anonymized biological samples of the biobank.

Note that full information on the approval of the study protocol must also be provided in the manuscript.

Field-specific reporting

Please select the one below that is the best fit for your research. If you are not sure, read the appropriate sections before making your selection.

☒ Life sciences ☐ Behavioural & social sciences ☐ Ecological, evolutionary & environmental sciences

For a reference copy of the document with all sections, see nature.com/documents/nr-reporting-summary-flat.pdf

Life sciences study design

All studies must disclose on these points even when the disclosure is negative.

Sample size	To calculate the required sample size for the Wilcoxon signed-rank test for matched pairs to test the difference in mosaicism between EM and CV , we assumed a normal parent distribution, a mean percentage of 43% mosaicism in the CV group, a standard deviation (SD) of 30 in both groups, and a correlation between the groups of 0.5. A total sample size of 35 participants was required to test a 15% difference between the EM and CV group, performing a two-sided test using an alpha of 0.05 and a power (1-β) of 0.80. The sample size calculation was performed using G*power 3.1.9.7.
Data exclusions	20 couples have been excluded from data analysis. For proper haplotyping of miscarried POC samples, adequate quality DNA is necessary from the parents as well as POC tissue. For 16 cases there was inadequate quality of DNA for at least one of the required samples. 4 couples were excluded due to one or both parental DNA samples having no parental match with the fetal tissues (not biological parents).

Replication	For 33 DNA samples, DNA quality was insufficient for proper haplotyping. For these samples, DNA was re-isolated and genotyped for analysis.
Randomization	The study participants were couples affected by pregnancy loss. From 1745 spontaneous pregnancy loss cases that were karyotyped, 111 families were randomly selected based on the following inclusion criteria: a normal karyotype, availability of parental blood samples and extraembryonic mesoderm and chorionic villi from the miscarried POC, and no identified genetic predisposition for pregnancy loss in the couple. For 91 families (94 POCs), a division was made between sporadic pregnancy loss (42 families and POCs), defined as 1 loss, and recurrent pregnancy loss (49 families and 52 POCs), defined as more or equal to 2 pregnancy losses.
Blinding	Prior to data analyses fetal and parental samples were anonymized. The investigators were blinded to group allocation during data collection.

Reporting for specific materials, systems and methods

We require information from authors about some types of materials, experimental systems and methods used in many studies. Here, indicate whether each material, system or method listed is relevant to your study. If you are not sure if a list item applies to your research, read the appropriate section before selecting a response.

Materials & experimental systems

n/a	Involved in the study
☒	☐ Antibodies
☒	☐ Eukaryotic cell lines
☒	☐ Palaeontology and archaeology
☒	☐ Animals and other organisms
☒	☐ Clinical data
☒	☐ Dual use research of concern

Methods

n/a	Involved in the study
☒	☐ ChIP-seq
☒	☐ Flow cytometry
☒	☐ MRI-based neuroimaging
